# Demethoxycurcumin Retards Cell Growth and Induces Apoptosis in Human Brain Malignant Glioma GBM 8401 Cells

**DOI:** 10.1155/2012/396573

**Published:** 2012-02-15

**Authors:** Tzuu-Yuan Huang, Che-Wen Hsu, Weng-Cheng Chang, Miin-Yau Wang, June-Fu Wu, Yi-Chiang Hsu

**Affiliations:** ^1^Department of Neurosurgery, Tainan Sin-Lau Hospital, Tainan 70142, Taiwan; ^2^Department of Nutrition and Health Sciences, College of Health Sciences, Chang Jung Christian University, Tainan 71101, Taiwan; ^3^Innovative Research Center of Medicine, College of Health Sciences, Chang Jung Christian University, Tainan 71101, Taiwan; ^4^Graduate Institute of Medical Science, College of Health Sciences, Chang Jung Christian University, Tainan 71101, Taiwan

## Abstract

Demethoxycurcumin (DMC; a curcumin-related demethoxy compound) has been recently shown to display antioxidant and antitumor activities. It has also produced a potent chemopreventive action against cancer. In the present study, the antiproliferation (using the MTT assay, DMC was found to have cytotoxic activities against GBM 8401 cell with IC_50_ values at 22.71 **μ**M) and induced apoptosis effects of DMC have been investigated in human brain malignant glioma GBM 8401 cells. We have studied the mitochondrial membrane potential (MMP), DNA fragmentation, caspase activation, and NF-**κ**B transcriptional factor activity. By these approaches, our results indicated that DMC has produced an inhibition of cell proliferation as well as the activation of apoptosis in GBM 8401 cells. Both effects were observed to increase in proportion with the dosage of DMC treatment, and the apoptosis was induced by DMC in human brain malignant glioma GBM 8401 cells via mitochondria- and caspase-dependent pathways.

## 1. Introduction

**Figure 1:**
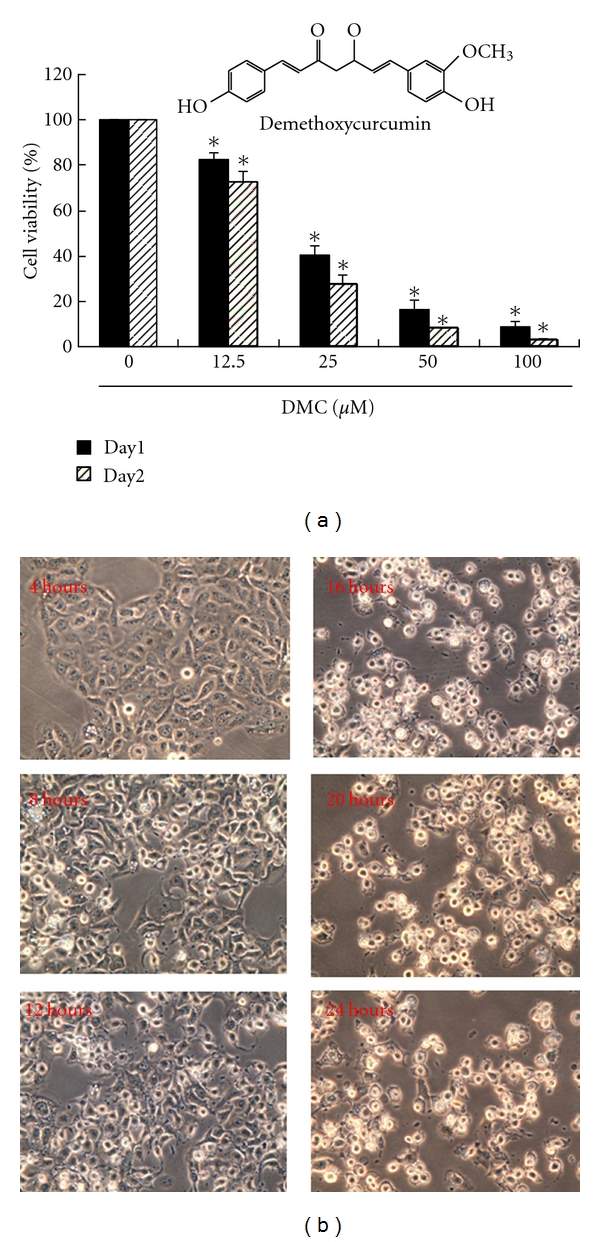
Demethoxycurcumin (DMC) mediates the survival of GBM 8401 cells and thus inhibits their proliferation. (a) In vitro study was initiated by treating each of the GBM 8401 cells to increasing doses of DMC (0, 12.5, 25, 50 and 100 *μ*M) for 24 or 48 hours. The survival of these DMC-treated cancer cells was then measured by MTT method. Results were expressed as a percentage of control, which was considered as 100%. All data were reported as the means (±SEM) of at least three separate experiments. Statistical analysis was via *t*-test, with the significant differences determined at the level of *P < 0.05 versus control group (DMC 0 *μ*M). (b) The morphology of the human GBM 8401 cells after the treatment with DMC (25 *μ*M) for 4 and 24 hours changed with the cells being distorted and suspended in the medium.

Turmeric (curcuminoids) is contained in curry (powder). It has been used extensively in Asian countries and also in traditional medicine [1]. Interest in this herb has grown in recent years based on its reported putative beneficial pharmacological effects, which include antioxidant, anti-inflammatory [2, 3], and cancer-preventive properties [4, 5]. In addition to the actions of inhibiting cell proliferation and increasing apoptosis [6–8], other mechanisms have also been proposed to rationalize the anticarcinogenic effect of curcuminoids (curcumin and its related demethoxy compounds: demethoxycurcumin (MW: 338.35 g/mol, Figure 1(a)) and bisdemethoxycurcumin (bDMC)), such as the anti-inflammatory and antioxidant activities, the induction of phase-II detoxification enzymes, the inhibition of cyclooxygenase 2 (COX-2), the effect on AP-1 and NF*κ*B transcription factors, the inhibition of matrix metalloproteinase (MMP) [9, 10], and the effect on protein kinases, others [11, 12].

A report showed that brain cancer is the eighteenth most common malignancy cancer in Taiwan [13]. However, the therapy for brain cancer is still not clear. One of the best strategies for tumor suppression is to induce the apoptosis pathway (caspase-dependent and -independent) in cancer cells [14]. The caspase-dependent pathway involves the death receptor and then promotes the activations of caspase 3, 8, and 9 [15]. The caspase-independent pathway is involved in mitochondria damage [16]. Many studies have demonstrated that chemotherapy drugs synthesized from plants repress tumor growth through the induction of apoptosis [17, 18]. Furthermore, other studies have linked chemoprevention and chemotherapy to the prevention of the downregulation of gap junctional intercellular communication by tumor promoters or the restoration of cell-cell communication in cancer cells [19]. Because modulation of gap junctional intercellular communication by epigenetic agents plays a major role in homeostatic regulation of both stem and progenitor cells in normal tissues, the modulation of this biological process by both endogenous and endogenous chemicals should be incorporated as an end point to monitor for potential toxicities or chemopreventive attributes [20]. In addition, apoptosis has also been linked to the upregulation of gap junction function [21, 22].

 Glioblastomas (GBMs) are highly lethal primary brain tumors, are grade IV gliomas, and appear to harbor therapy-resistant cancer stem cells (CSCs) that are the major cause of recurrence [23]. Human brain glioblastoma multiforms [24, 25] or malignant glioma [26] GBM 8401 cells were isolated and established from a Chinese female patient with brain glioblastoma multiforme (GBM) [27]. These are tumorigenic in athymic nude mice [28]. Recent studies have suggested that GBMs contain a subpopulation of tumor cells that displays stem cell characteristics and could therefore be responsible for in vivo tumor growth [29].Therefore, we chose the GBM 8401 cells as a human brain glioblastoma model to analyze the antitumor activity of DMC.

In this paper, studies have been initiated to investigate whether DMC could contribute to the antiproliferation and apoptosis of human brain malignant glioma GBM 8401 cells. We expect that all of these experiments could provide a scientific basis and technological support for brain glioblastoma therapy.

## 2. Materials and Methods

### 2.1. Cells Culture

The human brain malignant glioma GBM 8401 cells were purchased from the Food Industry Research and Development Institute (Hsinchu, Taiwan). These were maintained on culture dishes, in RPMI 1640 supplemented with 10% (v/v) FBS. The cells were cultured in an atmosphere containing 5% CO_2_ at 37°C incubator.

### 2.2. Cell Proliferation Assay

The cells were seeded into 96-well culture plates at 5000 cells/well. Different cell wells were treated with 0, 12.5, 25, 50, and 100 *μ*M DMC, respectively, for 1 or 2 days. MTT dye (1 mg/mL) was added to each well for the extra 4 hours after treatment. The reaction was stopped by the addition of DMSO, and optical density was measured at 540 nm on a multiwell plate reader (Powerwave XS, Biotek). Background absorbance of the medium in the absence of cells was subtracted. All samples were assayed in triplicate, and the mean for each experiment was calculated. Results were expressed as a percentage of control, which was considered as 100%. Each assay was carried out in triplicate and the results were expressed as the mean (±SEM). The change in cell morphology was determined microscopically by Olympus CKX41.

### 2.3. Mitochondrial Membrane Potential (MMP)

The GBM 8401 cells were first seeded in 24- or 6-well plates (Orange Scientific. E.U.). Following the treatment with DMC for 4 hours, Rhodamine 123 (10 *μ*g/mL, Sigma-Aldrich, St. Louis, MO, USA) and JC-1 (25 *μ*M) were added to the culture medium (500 *μ*L/well) and then incubated (37°C, 20 min) for mitochondria staining. After washing twice with a warm PBS, the cells were fixed with 2% paraformaldehyde, inspected by a fluorescence microscopy (Olympus CKX41 and U-RFLT 50), and the RFU (relative fluorescence unit) was detected by the BioTek FLx800 TBI. For Rhodamine 123, the wavelength settings were 504 nm and 534 nm. Each assay was carried out in triplicate, and the results were expressed as the mean (±SEM) of RFU and reported as the percentage of the RFU for the control group (DMC 0 *μ*M). For JC-1, the quantification by flow cytometry (BD FACScalibur, BD, USA) and mitochondria containing red JC-1 aggregates in healthy cells were detectable in the FL2 channel, and green JC-1 monomers in apoptotic cells were detectable in the FL1 channel.

### 2.4. Cell Cycle Analysis

The method for cell cycle analysis used propidium iodide (PI), that is, using the fluorescent nucleic acid dye PI to identify the proportion of cells that are in one of the three interphase stages of the cell cycle. The cells were treated with 0, 12.5, 25, and 50 *μ*M DMC for 24 hours, then harvested and fixed in 1 mL cold 70% ethanol for at least 8 hours at −20°C. DNA was stained in PI/RNaseA solution, and the cell cycle (at least 10,000 single cells) was detected by flow cytometry (FACSCalibur, BD, USA). Data was analyzed by WinMDI 2.8 free software (BD, USA). 

### 2.5. Measurement of Apoptosis

The GBM 8401 cells were first seeded in 6-well plates (Orange Scientific. E.U.). Following the treatment with DMC for 4 hours, the cells were harvested after the incubation period and washed in cold phosphate-buffered saline (PBS). A 1X annexin-binding buffer (BD Pharmingen, BD, USA) and 100 *μ*g/mL working solution of propidium iodide (PI) (Sigma, USA) were prepared. The washed cells were recentrifuged (the supernatant discarded) and resuspended in 1X annexin-binding buffer. Five *μ*L of FITC annexin V (BD Pharmingen, BD, USA) and 1 *μ*L of the 100 *μ*g/mL PI working solution were added to each 100 *μ*L of cell suspension, and the cells were incubated at room temperature for 15 minutes. After the incubation period, the stained cells were analyzed by flow cytometry, and the fluorescence emission measurement showed only low levels, apoptotic cells showed green fluorescence and dead cells showed both red and green fluorescence.

### 2.6. DNA Fragmentation Assay

The DNA fragmentation was detected by ApoAlert DNA fragmentation assay kit (Clontech, USA). The assay is based on terminal-deoxynucleotidyl-transferase-(TdT-) mediated dUTP nick-end-labeling (TUNEL). TdT catalyzes incorporation of fluorescein-dUTP at the free 3′-hydroxyl ends of fragmented DNA. The cells were treated with DMC for 16 hours, and the fluorescein-labeled DNA was detected via confocal microscopy system (CARV II, BD, USA) and flow cytometry (FACSCalibur, BD, USA); data were analyzed by WinMDI 2.8 free software (BD, USA).

### 2.7. Western Blot Assay

A total of 30–50 *μ*g proteins were separated by SDS-PAGE (10–12% SDS-polyacrylamide gel electrophoresis) and transferred to PVDF membranes (Millipore, USA) in a tank blotter (in 25 mM Tris/0.192 M glycine, pH 8.3/20% methanol) at 30 voltage overnight. The membranes were blocked with blocking buffer (Odyssey, USA) overnight and incubated with anti-*β*-actin (Sigma-Aldrich, St. Louis, MO, USA) and anti-caspase 3 (Santa Cruz BioTechnology, USA) antibody for 1.5*∼*2 hours. The blots were washed with Tris-HCl (pH 8.0/150 mM NaCl/0.05% Tween-20) for 3 × 10 minutes and incubated with second antibody (anti-rabbit or anti-mouse immunoglobulins) (IRDye Li-COR, USA) at 1/20000 dilution for 30 minutes. The antigen (*β*-actin and caspase 3) was then visualized by Odyssey near-infrared imaging system (Odyssey LI-COR, USA) and data analyzed by Odyssey 2.1 software.

### 2.8. Caspase Activity Assay

The caspase (2, 3, 8, and 9) activity was assessed by the ApoAlert Caspase assay plates (Clontech, USA). The cells were treated with DMC of 0, 12.5, 25, and 50 *μ*M with or without caspase-specific inhibitor for 8 hours. The caspase activity was detected by ApoAlert Caspase assay plates and inspected by the BioTek FLx800 TBI reader (BioTek, USA). The plates contained the fluorogenic substrates and inhibitors specific for different caspases. These substrates were covalently linked to their respectively activated caspases. The substrates were covalently linked to the fluorogenic dye 7-amino-4-methyl coumarin (AMC). Peptide-bound AMC excites in the UV range (380 nm) and emits at 460 nm. The AMC was normalized by total protein; each assay was carried out in triplicate, and the results were expressed as the mean (±SEM).

### 2.9. NF-*κ*B Transcription Factor Assay

The NF-*κ*B transcription factor was assessed by the NoShift II NF-*κ*B transcription factor assay kit (NOvagen, USA) and confocal microscopy. The cells were treated with 0, 12.5, 25, and 50 *μ*M DMC for 6 hours. After treatment, the cell nuclear fraction was isolated by NucBuster Protein Extraction Kit (NOvagen, USA). The NF-*κ*B transcription factor measures light intensity by microplate luminometer (BioTek FLx800 TBI reader BioTek, USA). The relative light units (RLUs) were normalized by total protein; each assay was carried out in triplicate, and the results were expressed as the mean (±SEM). Confocal microscopy was performed as described previously. Briefly, the GBM8401 cells (2 × 10^6^ cells) were treated with 0 and 25 *μ*M DMC for 6 hours and were fixed on coverslips. After treatment, samples were incubated with rabbit anti-human p50 antibody (SC-8414 PE, Santa Cruz Biotechnology) for 30 minutes then washed with PBS. The cells were mounted onto microscope slides using mounting medium containing DAPI.

### 2.10. Statistical Analysis

All data were reported as the means (±SEM) of at least three separate experiments. A *t*-test or one-way ANOVA with post hoc test was employed for statistical analysis, with significant differences determined as *P* < 0.05.

## 3. Results

### 3.1. DMC Inhibits the Proliferation of GBM 8401 Cells

It is hypothesized that DMC could mediate the survival of human brain malignant glioma GBM 8401 cells and thus inhibit their proliferation. To explore this antitumor activity of DMC against the GBM 8401 cells, an in vitro study was initiated by treating each sample of the GBM 8401 cells to increasing doses of DMC (0, 12.5, 25, 50, and 100 *μ*M) for 24 or 48 hours. The proliferation of these DMC-treated cancer cells was then measured by MTT method. The results summarized in Figure 1(a) indicate that the survival and proliferation of GBM 8401 cells were decreased by DMC treatment in a dose-dependent reduction manner. The IC_50_ of DMC in the GBM 8401 cancer cells was determined, respectively, to be 22.71 *μ*M; *y* = 88.413e − 0.0251x, *R* ^2^ = 0.921 (*P* < 0.05 versus DMC 0 *μ*M). Moreover, DMC was noted to induce a morphological change in the GBM 8401 cells. In Figure 1(b), microscopic examination shows that, following the exposure to DMC (25 *μ*M) for 4 to 24 hours, the cancer cells have displayed a remarkable change in their morphology. DMC induced the death of cancer cells, which formed a suspension in the medium.

### 3.2. DMC Reduces the MMP in GBM 8401 Cells

**Figure 2:**
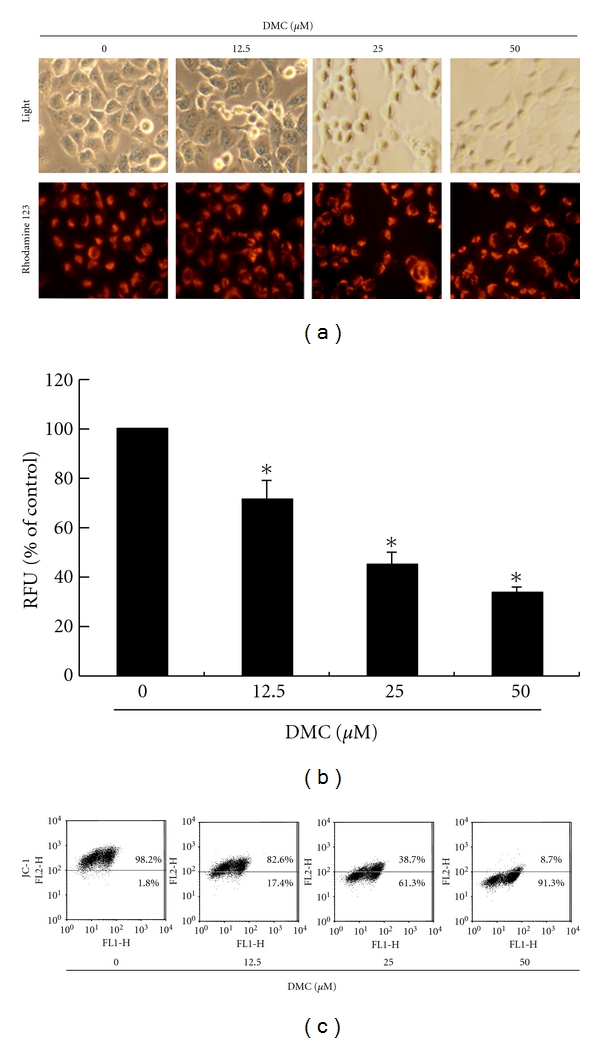
Reduction of the mitochondrial membrane potential (MMP) in the GBM 8401 cells by DMC, which was determined by Rhodamine 123 and JC-1 staining and detected by fluorescence microscopy, fluorescence reader, and flow cytometry: (a) MMP is shown to be significantly reduced in the GBM 8401 cells treated with DMC (12.5, 25, and 50, *μ*M) by Rhodamine 123 staining, and the same effect was also demonstrated by JC-1 staining (c). (b) Using BioTek FLx800 TBI fluorescence reader, the intensity of fluorescence was determined and found to decline (presented as the percentage of the controls) as the concentration of DMC used to treat the GBM 8401 cells increased. All the data shown are the mean (±SEM) of at least three independent experiments. The symbol (*) on each group of bars denotes that difference from the treatment with 0 *μ*M DMC is statistically significant at *P* < 0.05.

To explore the possible effect of DMC on the MMP in the GBM 8401 cells, Rhodamine 123 was used to determine the MMP in the DMC-treated cancer cells. The results compared in Figure 2(a) indicate that the MMP of the GBM 8401 cells has been reduced after treatment with DMC. The results summarized in Figure 2(b) indicate that the intensity of fluorescence, as determined by the BioTek FLx800 TBI fluorescence reader, decreases as the DMC dose increases. The observations imply that the reduction of MMP in the GBM 8401 cells depends on the dosage of DMC used (*P* < 0.05 versus DMC 0 *μ*M). The loss of mitochondrial membrane potential is a hallmark for apoptosis. It is an early event coinciding with caspase activation. In nonapoptotic cells, JC-1 exists as a monomer in the cytosol (green) and accumulates as aggregates in the mitochondria, which appear red. In apoptotic and necrotic cells, JC-1 exists in monomeric form and stains the cytosol green.  Figure 2(c) shows typical FL-1/FL-2 dot plots for JC-1 staining GBM8401 cells with and without apoptosis. DMC-free GBM8401 cells without apoptosis had red fluorescing J-aggregates. The green fluorescing monomers shown in the lower region indicate apoptotic cell lines (DMC 12.5, 25, and 50 *μ*M treatment). By flow cytometry assays, we observed that the cells exposed to DMC exhibited a dose-dependent decrease in JC-1 staining compared to the untreated control cells. This indicated that there was a loss of mitochondrial membrane potential in DMC-treated cells, which approached the loss of potential observed after treating the cells. As clearly observed from the figure, DMC induced significant depolarization at 25 and 50 *μ*M DMC concentrations wherein there was a 34- and 50-fold reduction in the ratio of red-green fluorescence intensity. Taken together, all these results suggest that DMC exhibited a potent antineoplastic effect in GBM 8401 cells through loss of mitochondrial membrane potential, ultimately leading to apoptosis.

### 3.3. DMC Treatment Induces Accumulated Sub-G0/G1 in GBM 8401 Cells

**Figure 3:**
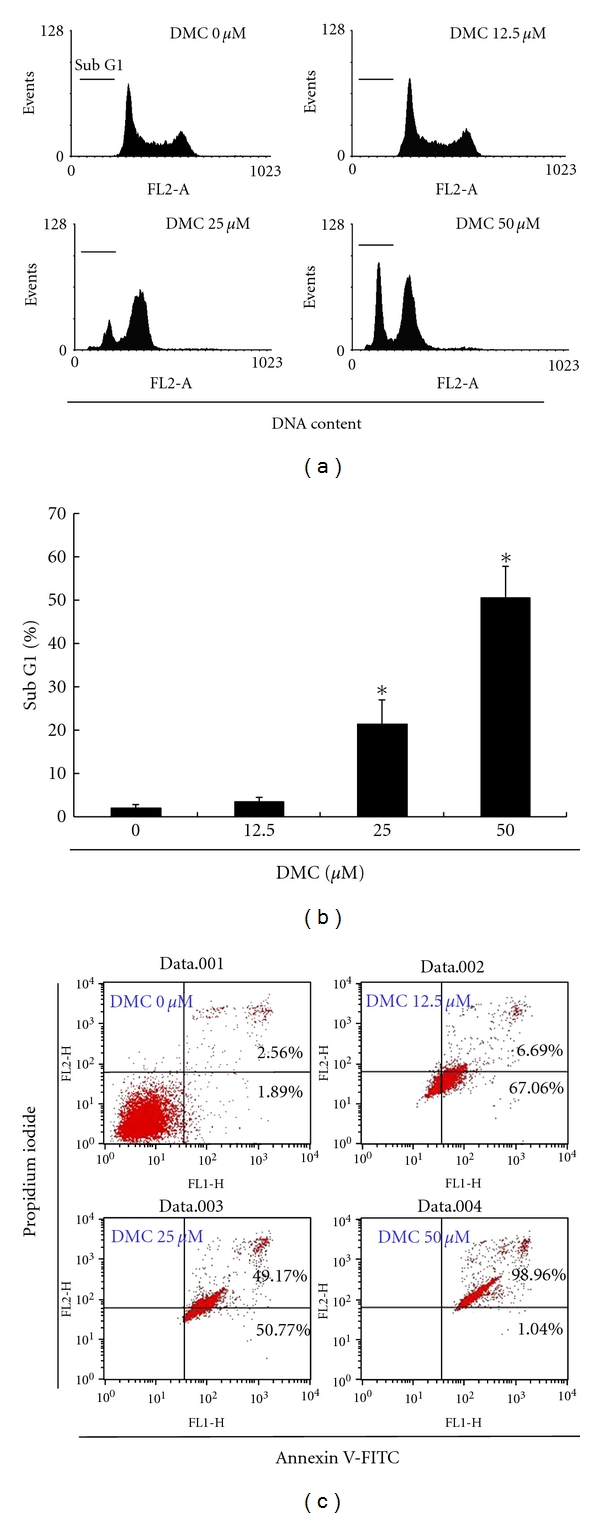
Effects of DMC on cell cycle progression/distribution and apoptosis in GBM 8401 cells. (a) cell cycle distribution and (b) cell cycle analysis of sub-G1 in GBM 8401 cells after culturing with DMC for 24 h. Treatment with DMSO (0.1%) was used as the control. All data were reported as the means (±SEM) of at least three separate experiments. Statistical analysis used the *t*-test, with the significant differences determined at the level of *P < 0.05 versus 0 *μ*M control group. (c) DMC induced the increase of both early apoptosis (Annexin V positive, PI negative) and late apoptosis/necrosis (Annexin V/PI double positive) in GBM 8401 cells for 4 h of incubation.

Cell-cycle distribution of DMC-treated GBM 8401 cells was analyzed by flow cytometry, aiming to determine whether the inhibitory effect was due to apoptosis. Before being processed and analyzed, the cells were exposed to DMC for a total of 24 hours. As shown in Figure 3(a), the cells exposed to DMC showed increase in the number of cells in the sub-G0/G1 phase, as compared with that of the untreated cells. The observations could imply that the GBM 8401 cells have undergone apoptosis. We found that treatment of DMC resulted in increase of cell populations in sub-G1 (Figure 3(b)) (*P* < 0.05 versus DMC 0 *μ*M) and a concomitant decrease of cell numbers at other phases (Figure 3(a)).

### 3.4. Induction of Apoptosis-Dependent Cell Death by DMC in GBM 8401 Cells

To further elucidate the anticancer mechanism of DMC in GBM 8401 cells, we performed apoptosis studies. After treating the cells with different doses of DMC, the percentage of apoptotic cells were assessed by Annexin V-FITC and propidium iodide staining, followed by flow cytometric analysis (Figure 3(c)). The dot plot of Annexin V-FITC fluorescence versus PI fluorescence also indicated a significant increase of the percentage of apoptotic cells that were treated by DMC. It was observed that, at concentrations of 12.5 to 50 *μ*M DMC, there was a significant increase in the percentage of cells undergoing apoptosis.

### 3.5. DMC Induced DNA Fragmentation in GBM 8401 Cells

**Figure 4:**
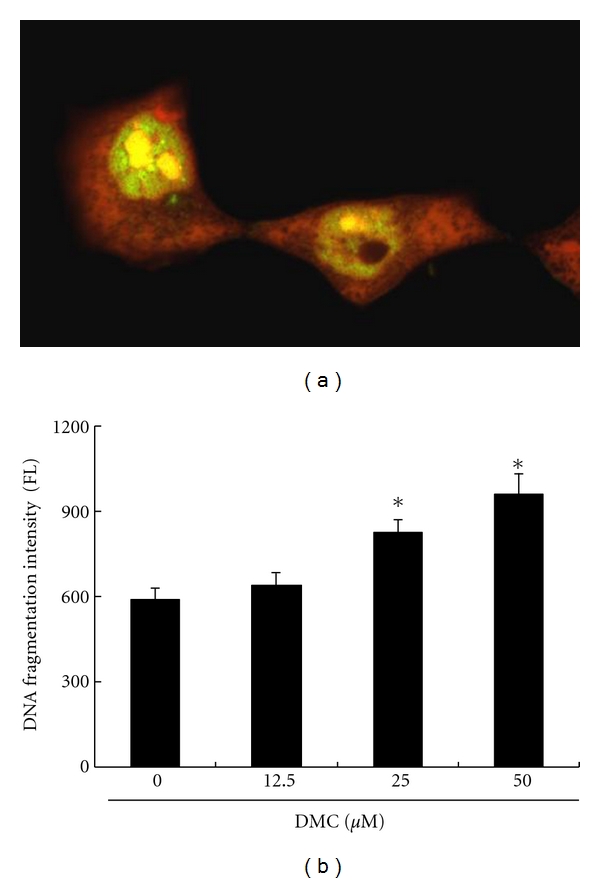
Demethoxycurcumin induced DNA fragmentation in GBM 8401 cells. (a) The cells were treated with DMC for 16 hours. The DNA fragmentation was detected by fluorescein-labeled DNA via confocal microscopy system. The apoptotic cells exhibit nuclear green fluorescence. All cells stained with propidium iodide exhibit red cytoplasmic fluorescence. (b) Quantification of DNA fragmentation by measuring the fluorescence intensities by flow cytometry. The data showed that DNA fragmentation levels were significantly elevated in cells incubated with DMC incubation for 16 hours. All data were reported as the means (±SEM) of at least three separate experiments. Statistical analysis used *t*-test, with the significant differences determined at the level of *P < 0.05 versus 0 *μ*M control group.

Cells undergoing apoptosis will lose part of their DNA (due to the DNA fragmentation in later apoptosis). The visibility of “sub-G1” peaks by flow cytometry might be an index of the formation of characteristic DNA ladders [30]. It is hypothesized that DMC could induce apoptosis of GBM 8401 cells via the DNA fragmentation. To explore this effect of DMC against the GBM 8401 cells, an in vitro study was initiated by treating each of the GBM 8401 cell samples with 25 *μ*M DMC for 16 hours. After treatment, the DNA fragmentation was detected by fluorescein-labeled DNA via confocal microscopy system and flow cytometry. The DNA fragmentation is illustrated in Figure 4(a), with apoptotic cells exhibiting nuclear green fluorescence. All cells stained with propidium iodide exhibit red cytoplasmic fluorescence. The results indicated that DMC induced DNA fragmentation in GBM 8401 cells. The quantification of DNA fragmentation was measured by the fluorescence intensities by flow cytometry (Figure 4(b)), showing that DNA fragmentation levels were significantly increased in cells incubated with DMC. Taken together, the observations imply that DMC significantly induced the DNA fragmentation of GBM 8401 cells.

### 3.6. Apoptosis Induction by DMC in GBM 8401 Cells via Caspase 3, 8, and 9 Activation

**Figure 5:**
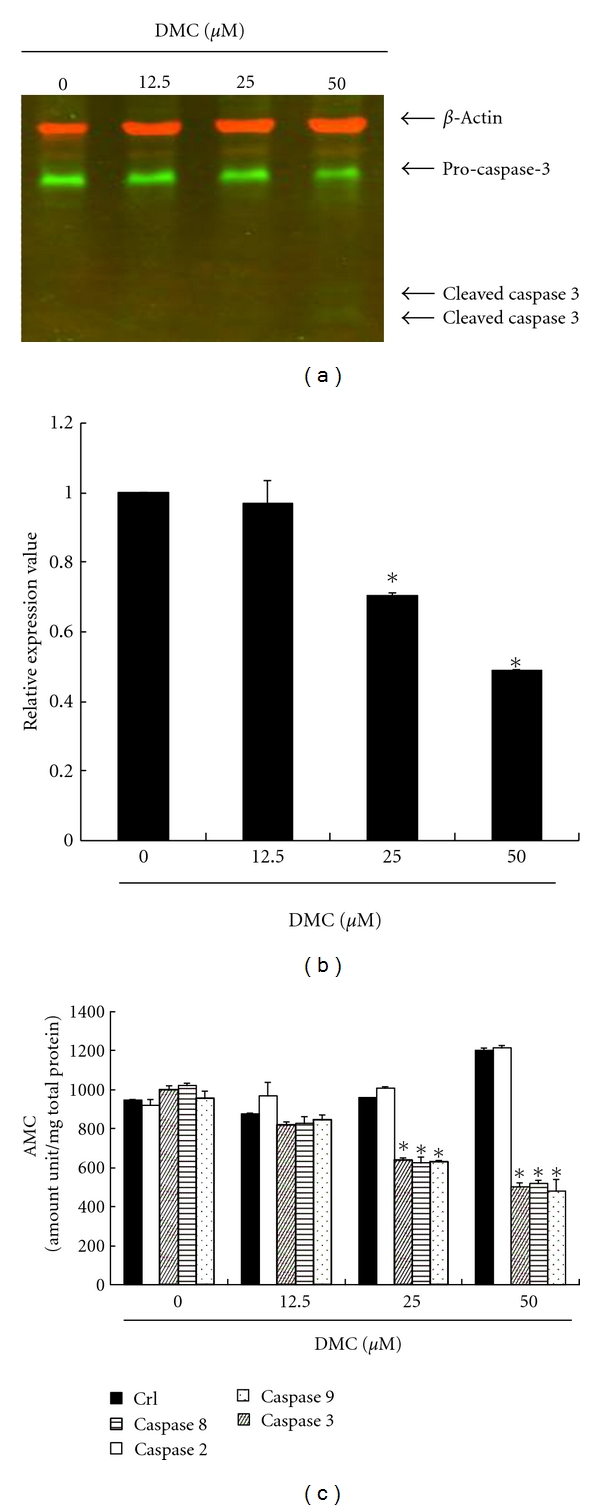
Apoptosis induction by DMC in GBM 8401 cells via caspase 3, 8, and 9 activations. DMC activated pro-caspase-3 degradation in GBM 8401 cells. The cells were treated with DMC (0, 12.5, 25, and 50 *μ*M), and then (a) Western blot analysis was performed for pro- and cleaved- caspase-3. (b) Quantification of band intensities by Li-COR near-infrared imaging system. All data were reported as the means (±SEM) of at least three separate experiments. Statistical analysis used the *t*-test, with the significant differences determined at the level of *P < 0.05 versus 0 *μ*M control group. (c) The caspase 2, 3, 8, and 9 activity was analyzed by ApoAlert Caspase assay plates. The DMC induced the caspase activity of GBM 8401 cells. All data were reported as the means (±SEM) of at least three separate experiments. Statistical analysis was as cited above.

Immunoblotting of cellular proteins from GBM 8401 cells treated with DMC showed decrease of pro-caspase-3 after DMC incubation (Figure 5(a)). Quantification of pro-caspase-3, done by measuring the relative band intensities, showed that pro-caspase-3 levels were significantly lower in cells incubated with DMC (Figure 5(b)). The results indicated that DMC induced caspase 3 activity via cleaved pro-caspase-3 and apoptosis after DMC incubation. As shown in Figure 5(c), the DMC elevated the caspase 3, 8, and 9 activities in GBM 8401 cells that had been decreased with caspase-specific inhibitors. The results summarized in Figure 5 indicate that the increased levels of caspase activity may play an important role in DMC-induced apoptosis in GBM 8401 cells.

### 3.7. DMC Inhibits Nuclear NF-*κ*B Transcription Factor Activity in GBM 8401 Cells

**Figure 6:**
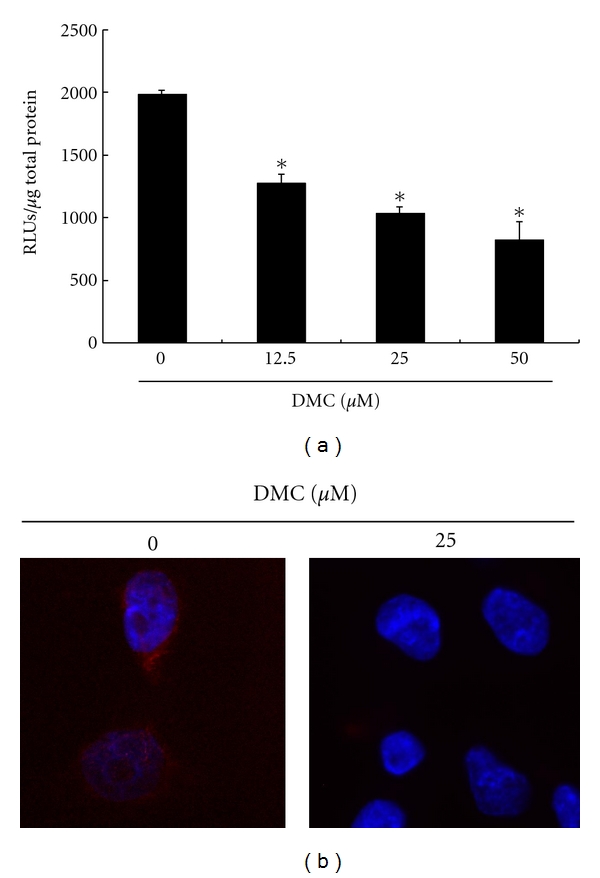
DMC inhibits nuclear NF-*κ*B transcription factor activity in GBM 8401 cells. (a) The NoShift II transcription factor assay kit was used to identify the activity of NF-*κ*B transcription factor in the cells after exposure to DMC followed by examination with microplate luminometer. (b) Diminished NF-*κ*B activity in DMC-treated GBM8401 cells. The cells were examined for their NF-*κ*B activity 6 hours after DMC stimulation by confocal microscopy of NF-*κ*B subunit p50/52 localization. The cells were stained for p50/52 (red). DAPI (blue) indicates nucleus, where an active form of NF-*κ*B subunit p50/52 is found. All data were reported as the means (±SEM) of at least three separate experiments. Statistical analysis was as cited above.

To explore the potential role where DMC inhibits nuclear NF-*κ*B transcription factor activity of GBM 8401 cells, the NoShift II transcription factor assay kit has been used to identify the activity of NF-*κ*B transcription factor in the GBM 8401cells after the 6 hours of exposure to DMC followed by examination with microplate luminometer. The results summarized in Figure 6(a) indicate that the NF-*κ*B transcription factor activity of GBM 8401 cells has been repressed through increasing the dose of DMC added into the cell cultures. The results in Figure 6(b) indicate that less NF-*κ*B subunit p50/52 was observed in the nuclei of GBM8401 cells treated with DMC 25 *μ*M than in the nuclei of DMC-free GBM8401 cells. The results could imply that the GBM8401 cells have had their activity of NF-*κ*B repressed in relation to increased dosage of DMC added into the cell cultures.

## 4. Discussion

Dietary constituents may display promising chemopreventive and chemotherapeutic potential and thus ameliorate the side effects associated with conventional chemotherapy [4]. Recently, more attention has been focused on complementary and alternative medicine (CAM) as an alternative therapeutic modality for treatment of cancer patients. Cell cycle progression and apoptosis are two pivotal signaling mechanisms used to maintain normal condition in healthy tissues. As a dietary supplement or spicing agent, curcuminoids are used worldwide and their many uses have led to studies aimed at elucidating the mechanism of their activities, in particular the anticancer activity [5]. Most anticancer agents and DNA damaging agents arrest the cell cycle at the G0/G1 or G2/M phase and then induce cell apoptosis. Our data from flow cytometry analysis showed that the cell cycle increased in the sub-G1 phase in GBM 8401 cells incubated with DMC. These results demonstrate an inhibitory role for DMC in GBM 8401 cells, which is associated with induction of apoptosis.

The results collected in this series of studies with the cell lines of human brain malignant glioma cells have provided experimental evidence to indicate that DMC could irreversibly induce apoptosis of these cancer cells. These culminate with several phase I human trials that have shown curcuminoids to be well tolerated [31]. The most common cell death mode on curcuminoids treatment seems to be apoptosis [32]. Apoptosis can be triggered by a large variety of different stimuli. To date, two major intracellular apoptosis signaling pathways have been identified: intrinsic and extrinsic. The intrinsic pathway involves an increase of outer mitochondrial membrane permeability. The extrinsic pathway involves ligation of death (Fas) receptor, resulting in the recruitment of the adaptor protein FADD through interaction between the death domains of both molecules [33]. In both pathways, the stress-mediated apoptosis is often triggered by mitochondrial function loss. Accordingly, this functional loss in DMC-mediated apoptosis was also explored in MTT viability and MMP assay. Therefore, the apoptosis induced by DMC was considered to be through the intrinsic pathway related to mitochondrial dysfunction.

Caspases, a family of cysteinyl aspartate-specific proteases, play an essential role in the regulation and the execution of apoptotic cell death. Multiple apoptotic stimuli trigger the activation of proteases called caspases; caspases are constitutively expressed in almost all cell types as inactive proenzymes that are processed and activated in response to a variety of proapoptotic stimuli [34]. All caspases are produced in cells as inactive zymogens and require a proteolytic cleavage and then convert to active form during apoptosis. Caspases are typically divided into three major groups, depending on the structure of their prodomain and their function [35]. The first is inflammatory caspases (1, 4, 5, 11, 12, and 14), the second group initiator-of-apoptosis caspases (2, 8, and 9), and the third effector caspases (3, 6, and 7) [36]. Caspase 2 was initially described as a neuronally expressed caspase that was downregulated during the course of brain development [37]. Two forms of caspase 2 were found—a short antiapoptotic form and a longer proapoptotic form [38]. It can be demonstrated that other caspases can replace caspase 2 as the executioner if inhibitors and facilitators of apoptosis are appropriately regulated. It is also clear that there exist many cell death paradigms where the executioner is not caspase 2, but rather caspase 3 or 7 [39]. Caspase 3 is the most extensively studied apoptotic protein and is a key effector caspase in the apoptosis pathway, amplifying the signal from initiator caspases (such as caspase 8) and signifying full commitment to cellular disassembly [40]. Caspase 8 is a key enzyme in the apoptosis pathway. Caspase 8 contains two N-terminal region death effector domains which are removed to activate the enzyme along with cleavage into the two subunits [41]. These subunits then form the active protease which is capable of cleaving caspase 3, 6, and 7, which correspondingly initiate the death cascade and finally induce apoptosis. Therefore, the DMC-induced apoptosis was mediated through activation of caspase cascade (3, 8, and 9).

Many studies have led to the discovery of two major apoptotic nucleases, termed DNA fragmentation factor (DFF) [42] or caspase-activated DNase (CAD) [43] and endonuclease G. Both endonucleases attack chromatin to yield 3-hydroxyl and 5-phosphate termini, first creating 50 to 300 kb cleavage products and then oligonucleosomal fragmentation, but these nucleases show different cellular locations and are regulated in fundamentally different ways. Although activation of the executorial caspases seems to be indispensable for realization of the apoptotic program, several forms of cell demise have been shown to be caspase-independent or even accelerated by caspase inhibitors [44]. The observations of this study have implied that DMC has significantly induced the DNA fragmentation of GBM 8401 cells.

Nuclear factor-*κ*B (NF-*κ*B) plays an important role in cell proliferation and apoptosis by regulating the expression of genes involved in these processes [45]. Active NF-*κ*B is most commonly composed of the heterodimer DNA-binding subunits p50 and p65. It has recently been shown that inactivation of p65 subunit of NF-*κ*B leads to the death through apoptosis of liver cells [46]. Similarly, it has been shown in a wide range of cells that when NF-*κ*B has been inactivated by I*κ*-B*α*, cells were more sensitive to TNF-*α*—induced apoptosis. Evidence exists for NF-*κ*B playing both anti- and proapoptosis roles [47]. The release of NF-*κ*B facilitates its translocation to the nucleus, where it promotes cell survival by initiating transcription of genes encoding stress-response enzymes, cell-adhesion molecules, proinflammatory cytokines, and antiapoptotic proteins [48]. The reducing levels of NF-*κ*B may be involved in curcuminoid-induced apoptosis of GBM 8401 cells.

Curcuminoids, well-established dietary antioxidants, are a safe natural food coloring additive with lipid-lowering potency in vivo [49, 50] and anticarcinogenic [51, 52], hepatoprotective [53], and neuroprotective properties [54], against heavy-metal-induced neurotoxicity and Alzheimer's disease [55, 56]. Curcuminoids have shown a variety of biological activities for various human diseases in preclinical setting. Thier poor oral bioavailability poses significant pharmacological barriers to thier clinical application. Liposomal [57] or nanoemulsion curcuminoids (NECs) [58] may conducted the pharmacokinetics of curcuminoids in vivo [59]. Purkayastha et al. show that a soluble formulation of curcuminoids crosses the blood-brain barrier but does not suppress normal brain cell viability. Furthermore, tail vein injection, or more effectively intracerebral injection through a cannula, blocks brain tumor formation in mice [60] or has neuroprotective effect on focal cerebral ischemic rats [61].

Taken together, in this work, the bioactivities of DMC were studied. In particular, it could effectively induce the apoptosis and cell cycle sub-G1 and cell cycle arrest of GBM 8401 cells. The apoptosis induction was revealed to be through activation of the caspase cascade. These results indicate that DMC is deserving further study as a potential chemotherapeutic drug.
